# Altered Adipokine Expression in Tumor Microenvironment Promotes Development of Triple Negative Breast Cancer

**DOI:** 10.3390/cancers14174139

**Published:** 2022-08-26

**Authors:** Efthymia Papakonstantinou, Zoi Piperigkou, Nikos K. Karamanos, Vasiliki Zolota

**Affiliations:** 1 Department of Gynecology and Obstetrics, School of Medicine, University of Patras, 26504 Patras, Greece or; 2 Biochemistry, Biochemical Analysis and Matrix Pathobiology Research Group, Laboratory of Biochemistry, Department of Chemistry, University of Patras, 26504 Patras, Greece; 3 Foundation for Research and Technology-Hellas (FORTH), Institute of Chemical Engineering Sciences (ICE-HT), 26504 Patras, Greece; 4 Department of Pathology, School of Medicine, University of Patras, 26504 Patras, Greece

**Keywords:** adipokines, adipose, cytokines, triple negative breast cancer, TNBC

## Abstract

**Simple Summary:**

Obesity is highly associated with overall risk for breast cancer and correlated with aggressive behavior. Although recent advances indicated that increased BMI may be an important factor for triple negative breast cancer development, the molecular mechanisms have not been fully elucidated. Obesity predisposes to breast cancer via metabolic modifications of tumor microenvironment. Altered adipocyte accumulation results in secretion of inflammatory factors, chemokines and adipokines which establish a pro-tumorigenic microenvironment that stimulates breast cancer induction, evolution and metastasis and impede anti-cancer drug response. The present article focuses on the association between adipokine expression and breast cancer, particularly triple negative subtype, and provides a summary and critical presentation of novel research findings. The main effort is to better determine strategies for tumor stratification and management.

**Abstract:**

Obesity is a remarkably important factor for breast carcinogenesis and aggressiveness. The implication of increased BMI in triple negative breast cancer (TNBC) development is also well established. A malignancy-promoting role of the adipose tissue has been supposed, where the adipocytes that constitute the majority of stromal cells release pro-inflammatory cytokines and growth factors. Alterations in adipokines and their receptors play significant roles in breast cancer initiation, progression, metastasis, and drug response. Classic adipokines, such as leptin, adiponectin, and resistin, have been extensively studied in breast cancer and connected with breast cancer risk and progression. Notably, new molecules are constantly being discovered and the list is continuously growing. Additionally, substantial progress has been made concerning their differential expression in association with clinical and pathological parameters of tumors and the prognostic and predictive value of their dysregulation in breast cancer carcinogenesis. However, evidence regarding the mechanisms by which adipose tissue is involved in the development of TNBC is lacking. In the present article we comment on current data on the suggested involvement of these mediators in breast cancer development and progression, with particular emphasis on TNBC, to draw attention to the design of novel targeted therapies and biomarkers.

## 1. Introduction

Many clinical and epidemiological studies published during the last decade have examined the correlation between obesity and breast cancer. It has been conclusively proved that obesity is a highly significant risk factor for breast carcinogenesis, cancer progression, and aggressiveness [1].

It has been proposed that overweight status increases breast cancer incidence in postmenopausal women by 30 to 50% [2], while in pre-menopausal women the evidence is still controversial [3]. As for post-menopausal women, obesity is primarily responsible for breast cancer that is positive for both hormonal receptors (ER+/PR+) and there is a linear correlation between the waist circumference and the likelihood of developing breast malignancy [4,5]. ER negative breast cancer and TNBC are marginally or conversely associated with obesity after menopause [5].

In contrast, obesity is connected to a reduced risk for the evolution of HR-positive breast cancer in premenopausal women but an increased risk for HR-negative, basal-like, and triple negative breast cancer (TNBC) [5,6]. Accumulative data suggest that increased body mass index (BMI) may be a significant factor for TNBC development and the frequency of obese TNBC patients is greater in comparison with that of non-obese patients [7]. Obese BMI is also a robust risk factor for inflammatory breast carcinoma, independently from menopausal and estrogen receptor status [8].

In both breast cancer patient groups (pre- and post-menopausal), high BMI is correlated with poorer survival, elevated recurrence risk and mortality, as well as ominous clinicopathologic features such as tumor size, grade, and lymph node metastases [9]. Indeed, overweight women with BMI ≥ 40.0 who suffer from breast cancer are three times more likely to die from breast cancer compared to the women with a BMI index below 21 [10]. Obese TNBC patients also tend to have larger tumors, higher T stage, and higher tumor grade [11]. Weight gain post diagnosis is also correlated with heightened breast cancer-specific mortality as well as with greater incidence and severity of surgery complications [12,13].

Researchers have also underlined the correlation between diabetes and breast cancer progression and prognosis, since it is a risk factor regarding distance metastasis evolvement [14,15,16]. Essentially, all of the adipocyte-associated clinical situations may be regarded as responsible factors for cancer initiation and/or progression [17,18]. Nevertheless, how changes in the metabolic status of the adipose tissue may alter tumor evolution remains ambiguous.

All cumulative data converge to suggest that the obesity epidemic has resulted in increased incidence of several cancer types, including breast cancer [2,19], via metabolic modifications that take place in the tumor microenvironment (TME) that promote tumorigenesis [20,21].

## 2. Obesity Fat Tissue and Tumor Microenvironment

Adipose tissue assumes the role of a primary element of the tumor microenvironment (TME). The cooperation among adipocytes and tumor cells has been broadly explored. Cancer-associated adipocytes (CAAs) are situated neighboring to cancer cells and interact with them through paracrine and endocrine effects [22]. The interplay between adipocyte and cancer cell affects both cell types phenotypically and functionally and initiates tumor development [23]. The gain of a CAA phenotype is considered an adjustable characteristic of adipocytes that react to stimuli from the TME [22,23].

Extracellular matrix (ECM) macromolecules are also a significant partner of TME. Advances in the field of ECM effectors related to breast cancer have been recently described [24,25]. Disproportionate adipose tissue in obese patients not only modifies the regional status by causing ECM remodeling but also supports breast cancer cell proliferation, immune evasion, and chemotherapy resistance by providing both energy and niches [26,27].

In human breast stroma, adipocytes display the most plentiful cell type and that explains the association between adipose tissue aggregation and tumor growth, via release of pro-inflammatory cytokines and growth factors [28,29,30].

“Adiponcosis” is the term that has been assigned to the notion that adipose tissue is involved in cancer development [31].

As in breast cancer [5], and in several other tumors including prostate [32], ovarian [33], and colon [34] cancer, cancer obesity designates ominous consequences for the patient.

Adipose tissue secretes several bio-active molecules, with endocrine activities, including inflammatory cytokines, peptide hormones, exosomes, miRNA, etc., which are sufficient to mediate systemic effects and regulate numerous physiological and pathological processes [35]. Obesity-associated adipocyte aggregation results in an altered profile of such mediators that establishes the peritumoral environment and advocate tumorigenesis via an adipocyte–cancer cell paracrine loop [36].

CAAs are in an active state attributable to the excretion of the chemokines CCL2 and CCL5, the cytokines IL-1b, IL-6, and TNFa, as well as the matrix metalloproteinase MMP-11 [29]. The CAA phenotype is highly associated with the release of several metabolites (ex. Pyruvate, free fatty acids, and ketones) [37]. Such state resembles the hypoxic situations that result in immunosuppression, in part through upregulation of HIF-1a and c-myc [38,39,40]. As far as their localization is concerned, the cells are detected near the invasive front of cancer cell aggregates [29,41].

The adipokines possess pro- and anti-inflammatory effects and play an essential role in energy homeostasis and immune system regulation [42]. Throughout the development of obesity, the release of adipokines with pro-inflammatory activities (leptin, resistin, IL-1, IL-6, IL-8, TNF-a) and the downregulation of anti-inflammatory ones (adiponectin, IL-10) establish a chronic state of inflammation, stimulated by NF-κΒ that predispose to metabolic morbidities (insulin resistance, type 2 diabetes and cardiovascular disorders) and cancer [43,44]. More specifically, the inflammatory changes caused by excessive fat accumulation cause extensive remodeling of the adipose tissue microenvironment such as fibrosis and angiogenesis leading to pro-oncogenic environment and acquisition of tumorigenic properties [45].

Although the role of excess fat accumulation in breast cancer pathogenesis is not fully understood, recent evidence underlines the role of hormonal alterations, insulin resistance, and deregulated secretion of adipose cytokines (adipokines) in obese, particularly post-menopausal, women with breast cancer [46]. It is well known that adipose tissue exerts highly complex endocrine activities and regulates the production of bioactive sex hormones, which increase breast cancer risk. More specifically, adipose tissue releases aromatase enzymes that transform androgens to estrogens and induce hyperinsulinemia and elevated IGF-1, which increase free estradiol and testosterone [47]. Estradiol has the ability to control the expression and activity levels of major ECM components via ERα axis [48]. In breast cancer, adipokines secreted by breast adipose tissue bind to their receptors on tumor cells and mediate cancer initiation and progression [49].

To date, several adipokines have been linked with breast cancer and the list is continuously growing. Some of them are exclusively secreted from adipose tissue (i.e., leptin, resistin, and visfatin), whereas others are secreted from other tissues as well. The expression of leptin, adiponectin, and resistin, which represent classic adipokines, and their differential expression in association with clinicopathological parameters have been largely studied in breast cancer [50]. However, little certainty exists regarding the precise involvement of these suggested mechanisms in triple negative breast cancer.

The aim of the present review was to provide a summary of data concerning adipokine expression in breast cancer, giving special emphasis to TNBC and providing all the current knowledge about mechanisms that underlie adipose tissue associated pathways with TNBC development, in an effort to draw attention to novel targeted treatments.

## 3. TNBC and Adipose Tissue

The cross talk linking adipose tissue and TNBC cells has been explored in many in vitro studies. Working with breast cancer cell lines as well as xenografts it was demonstrated that adipose tissue stem cells (ASCs) promoted tumor growth, epithelial mesenchymal transition (EMT), and invasion of breast cancer cells [51,52,53,54,55] and altered the composition of ECM [56,57].

Probable mechanisms by which ASCs might increase the metastatic capacity of breast cancer cells include initiation of EMT, matrix degradation through elevated expression of MMPs, induction of angiogenesis, and secretion of paracrine factors favoring metastatic spread [51], PDGF-D [53], IL-6 [58], and IL-8 [52].

Recently, in three-dimensional (3D) culture studies, the interaction of adipocytes with triple negative tumor cells was demonstrated. In the presence of ECM, which is rich in laminin, mature adipose cells can generate mesenchymal epithelial transition (MET) in the MDA-MB-231 and HS478t TNBC cell lines but not on SUM159 (mixed phenotype) or MCF-7 (epithelial phenotype) cells [59]. The authors suggested that one possible mechanism through which obesity is involved in tumor progression is the interplay of adipocytes with tumor cells and the development of secondary tumors through the MET mechanism.

Studies have also associated excess adiposity and high blood glucose levels to Basal-Like Immune Activated subtype of TNBC along with inflammatory and immunity-related factors such as CCL5 which explain poor cancer prognosis in metabolically unbalanced individuals [60].

Although data argue that obesity thoroughly changes the biology of TNBC, the pathways that link obesity with TNBC risk have not been fully clarified. It has been postulated that elevated amounts of sex steroids, 17HSD1 haplotypes, leptin, adiponectin, insulin, and IGF-I associated with increased BMI contribute to the development of TNBC [7].

In the next paragraphs, the specific interactions and involvement of each of the best-known as well as novel adipokines in TNBC are discussed.

## 4. Adipokines and TNBC

### 4.1. Leptin

Leptin is a polypeptide hormone, primarily produced by subcutaneous adipose tissue. Leptin was detected by cloning of the obesity (ob) gene [61] and is involved in satiety, food intake energy adequacy, and regulation of body weight [62]. Circulating levels of leptin increase along with increase of adipose tissue mass.

LEP acts via binding to its receptor (LEPR), which is a ubiquitously expressed, single transmembrane protein. Leptin receptor stimulates phosphorylation of the Janus kinase 2 (JAK2) and insulin receptor substrate-1 (IRS-1); activates STAT3 and 5; and induces ERK and PI3/Akt [63]. Obesity is associated with hypoxia which induces LEPR expression [64]. In cancer tissues, data suggest that LEPR activates similar oncogenic mechanisms and promotes glucose metabolism cell proliferation, angiogenesis, inflammation, and invasion [65]. Leptin and LEPR are expressed in many tumors such as breast, colon, prostate, and brain [66].

Leptin contributes to breast carcinogenesis through the increase of peripheral estrogen levels (directly or through upregulation of aromatase) and a positive association between serum leptin and estradiol has been found in pre- and post-menopausal women with breast cancer [67]. Clinical data have also confirmed the correlation between high serum leptin and breast cancer. In a recent meta-analysis leptin was demonstrated to be a significant risk factor for breast cancer in overweight/obese post-menopausal women [68]. High plasma leptin levels were also correlated with high grade, stage, and HR negativity [69]. In human breast cancer, elevated LEPR expression is also associated with poor prognosis [66,70].

In breast cancer leptin and LEPR expression is higher in tumor cells than in the normal breast epithelial cells and associated with tumor size and high tumor cell proliferation [71]. Additionally, in obese breast cancer patients, leptin expression has been reported to be higher in the adipose tissue neighboring the tumor than in the adipose tissue far from the tumor cells.

It has been widely suggested that obesity heightens the possibility of TNBC development [7]. In ΤΝΒC specimens, leptin and LEPR were significantly overexpressed [72]. A significant interplay among leptin and IGF-I signaling has been suggested in cancer cells which synergistically increase the activation of EGFR and LEPR and mediate TNBC progression [73].

In vivo studies in mice (MMTV-Wnt-1) have shown elimination of tumor growth as well as breast cancer stem cell population suppression after leptin signaling inhibition [74]. In particular, leptin induces breast cancer stemness and resistance to chemotherapy [75], through complex mechanisms that comprise signaling cross talk between Notch, IL-1, and leptin (NILCO) [76] and epigenetic downregulation of miR-200c in TNBC cells [77].

It has been previously shown that expression of LEPR in breast cancer cells is responsible for maintaining CSC-like and metastatic properties. *LEPR* suppressed MDA-MB-231 cells exhibited a MET phenotype with reduced cell growth and suppression of stem cell transcription factors *NANOG*, *SOX2*, and *OCT4*. It was concluded that *LEPR* inhibition might be a successful therapeutic approach to abrogate *NANOG* and CSC activities [70]. It was also indicated that the peptide Allo-aca, an antagonist of leptin receptor, inhibited proliferation in MDA-MB-231 cell line that was induced by leptin and significantly extended the average survival time of MDA-MB-231 mouse xenograft model and could become a treatment of choice for TNBC [78].

Recently it was suggested that increased levels of leptin secreted by obesity altered adipose stem cells induced the EMT and CSC reprogramming of MCF7, BT20, HCC1806, and TU-BcX cancerous cells through expression of Serpine1, SNAI2, IL6, TWIST1, and PTGS2. It was concluded that the obesity altered microenvironment conferred enhanced metastatic potential upon TNBC cells through leptin-mediated pathways [79].

Although leptin and its receptor appear to be promoters of malignancy, there are studies showing that they may have the opposite effect in certain circumstances. It was reported that decreased immunohistochemical expression of LEPR was associated with ER status and triple negativity and suggested that lower expression of the LEPR (rather than higher expression as hypothesized) is an important biomarker of breast cancer associated with aggressiveness, separated from race, BMI, and menopausal status [80].

Another group indicated that leptin induces apoptosis in TNBC cells when used with cAMP elevating agents [81]. This synergistic interaction between leptin and cAMP composites permits a decline in the efficacious doses of cAMP elevating drugs in vitro, thus conceivably reducing their offensive effects in vivo.

Further research is needed that examines leptin, LEPR, and their downstream effectors as future targets for precision medicine in obese TNBC patients.

The results from the literature relating to the expression of adipokines with pro-tumor activity in TNBC, including leptin, are summarized in Table 1.

### 4.2. Adiponectin

Adiponectin is the most abundant adipokine, composed of 244 amino acids and secreted exclusively by mature adipocytes [103]. It exerts its biological effects through binding with the receptors, AdipoR1, AdipoR2, and T-cadherin [104]. Serum levels of adiponectin, that tend to be higher in women than men, are influenced by several factors, including hormones, diet, and drugs [105]. Adiponectin affects several metabolic processes such as insulin secretion regulation and functions against inflammation and atherogenesis [106]. Lower adiponectin levels are associated with obesity, type 2 diabetes, and metabolic syndrome [107,108]. Adiponectin also demonstrates anti-proliferative, anti-migratory, and pro-apoptotic properties and, in contrast with leptin, influences negatively breast tumorigenesis [109]. It is inversely associated with serum glucose and leptin [110].

When adiponectin links to its receptor it activates the AMPK/LKB1 axis which is associated with cell proliferation, apoptosis, angiogenesis, and cellular metabolism. These actions are exercised by inhibition of MAPK, PI3K/Akt, WNT-β-catenin, NF-κB, and JAK2/STAT3 pathways [111].

The most recent meta-analysis found that breast cancer patients had lower serum adiponectin levels irrespective of menopausal status [112]. Others found an association only in post-menopausal patients [113]. Adiponectin expression in breast cancer tissue is lower in obese women, when compared with normal BMI and overweight ones [114]. Expression of the receptors AdipoR1 and AdipoR2 is also deregulated in breast cancer [115]. Thereby, adiponectin plays an opposite role compared to leptin in breast cancer progression, but the results greatly depend on their ER status. Recent data suggest that an elevated leptin:adiponectin ratio or, vice versa, a diminished adiponectin:leptin one, better correlates with TNBC rather than HR positive breast [116].

In ER/PR-negative breast cancer cells, adiponectin inhibits cell growth, invasion, migration, and vascular proliferation and induces apoptosis and autophagic cell death [117,118]. Normal adiponectin amounts significantly suppress the proliferation of MDA-MB-231 cancer cells [119], whereas decreased adiponectin is strongly correlated with TNBC development and progression, regardless of obesity and insulin resistance [120].

However, the results are conflicting when examining its reactions on ER-positive breast cancer cells. Specifically it seems that adiponectin may provoke ERα-positive breast cancer cell growth via heightened aromatase activity and production of local estrogens [121].

Conclusively, studies indicate that adiponectin exposure differently influences cell growth in ERa-negative and ERa-positive breast cancer and several issues remain to explain in detail.

Table 2 summarizes the results from the literature relating to the expression of adipokines with anti-tumor activity in TNBC.

### 4.3. Resistin

Resistin is an hormone secreted from adipocytes that is associated with obesity, insulin resistance, and diabetes type 2 [124]. Resistin exists in three isoforms: RELM-a, β, and γ [125]. Resistin activates MAPK, PI3K, p38, and NF-kB pathways through binding toTLR4 and promote cancer cell proliferation and migration [126,127]. No specific receptors for resistin have been investigated. High expression of resistin has been associated with visceral obesity, coronary artery disease, lung disease, and various cancers including breast, endometrial, and colorectal [125].

In the context of breast cancer, it was discovered that resistin increases the metastatic potential of MCF-7 and MB-231 human breast cancer cells by stimulation of EMT and stemness [82,83]. Resistin concentrations in breast cancer cells were also associated with cancer stem cell survival as well as invasion and migration via EMT [83,128].

Recently, it has been described that high resistin expression in breast cancer cells confers resistance to chemotherapy through suspension of doxorubicin-induced apoptosis [129]. Others also indicated that the high fat diet (HFD) attenuated the treatment efficacy of doxorubicin through elevation of both leptin and resistin [130]. In spite of all this, how excessive fat stimulates adipocytes to initiate downstream cytokine reaction and induce breast cancer remains largely unknown.

In a recent study it was revealed that diet-induced obesity causes overexpression of the TAZ in the adipocytes through the FFA/PPARγ axis. Knockdown of the adipocytic TAZ in mice downregulated the secretion of resistin and inhibited breast cancer proliferation and stemness [84]. Tumor samples from TNBC patients immunostained for both TAZ and resistin showed that higher expression of both molecules in adipose tissue correlated with higher stage and poor prognosis, giving the idea of targeted therapy.

Clinical data from independent groups link resistin expression in breast cancer tissue with adverse clinical and pathological characteristics as well as decreased survival [131]. Subsequent meta-analyses have shown significantly higher resistin levels in breast cancer patients but failed to prove a strong diagnostic and predictive value [132].

In a comprehensive differential survival analysis from the Cancer Genome Atlas (TCGA), resistin was elevated more than four times in breast cancers from African American patients. The study suggested that its presence at such high expression levels may indicate that insulin resistant type 2 diabetes and obesity, strongly linked with resistin, may play a crucial role in determining breast cancer development in AA women [133].

### 4.4. NAMPT/Visfatin

NAMPT, formerly noted as PBEF (pre-B cell colony enhancing factor), is an enzyme which exists in two forms, the intracellular iNampt and the extracellular-eNampt [134]. iNampt is responsible for most of the NAD+ biosynthesis in mammals and eNampt is a cytokine that bind and activates TLR4. Nampt/PBEF was lately re-named as visfatin a “visceral-fat-derived-hormone”, because it is excreted in the visceral fat and its circulating levels are elevated in obesity [135].

It has been described that visfatin binds to the insulin receptor and lower plasma glucose levels in cultured cells; however, its exact role as insulin mimicking cytokine is still under investigation. In cancer, including breast cancer, visfatin induces inflammation and immunosuppression, mainly through NF-kB regulation [136] as well as cell proliferation and apoptosis inhibition through AKT/PI3K and ERK/MAPK signaling [137]. Several papers have indicated that serum visfatin levels are upregulated in breast cancer [138,139] and associated with worse prognosis and aggressive behavior [140]. Accordingly, higher visfatin expression in breast cancer tissue is correlated with ER and PR negativity, ominous features and poor survival [141]. Another group found that high serum visfatin levels were associated with c-Abl and STAT3 activation. Inhibition of both c-Abl and STAT3 suspended cell proliferation and metastatic potential [139].

In the context of TNBC, a recent study has taken further steps towards a better understanding of the significance of NAMPT in TNBC biology. It was described that NAMPT-AS recruited POU2F2 to the promoter region of NAMPT, which regulated its expression both at the transcriptional and pos-transcriptional level [85]. Subsequently both axes triggered mTOR pathway, suppressed autophagy and apoptosis and assisted cell survival and invasiveness.

NAMPT knockdown in mice remarkably reduced the tumor growth, whereas further examination of NAMPT by immunohistochemistry in 480 human breast cancer tissues, with tumor microarrays (TMA), showed that patients with elevated NAMPT expression, whether belonging to the TNBC group or not, had ominous prognosis. Conclusive remarks suggested that NAMPT may serve as a prognostic marker and NAMPT-AS/NAMPT as an emerging curative target for TNBC in the near future.

In another study, carried out for the better understanding of the mechanisms underlying the response of TNBC patients to PARP inhibitors, the activity of the enzyme β-NAD(+) was investigated, which is involved in the metabolism of PARP [86]. It was identified that NAMPT participates in the generation of β-NAD (+) and was considered as an essential modifier of olaparib response. Interestingly, the NAMPT inhibitor FK866, when used in combination with olaparib inhibited the growth of TNBC cells, to a greater extent that when used each agent separately, justifying the combined use of NAMPT and PARP inhibitors for the treatment of TNBC.

Lately it was established that the molecule KPT-9274, which inhibits both PAK4 and NAMPT, abrogates the proliferation of TNBC cells and leads to cell death [87]. RNA sequencing of TNBC cells treated with KPT-9274 revealed Rictor, a component of mTORC2, as a crucial point. The authors suggested that the KPT-9274 may block cell growth, via inhibition of mTORC2 signaling.

Others reported significant restraint of HCC1806 and MDA-MB-231 cells growth, determined by a cell viability assay, after treatment with FK866 (a Nampt inhibitor), FX11 (lactate dehydrogenase A inhibitor) and paclitaxel [88]. The combinations of the drugs were subjected to optical redox imaging (ORI) and the results were correlated with the intracellular levels of reactive oxygen species (ROS).

The results of NAMPT/visfatin expression and TNBC are presented in Table 1.

### 4.5. Lipocalin-2

*Lipocalin*-2 (LCN2), which is also known as NGAL (neutrophil gelatinase-associated lipocalin), binds to and transports small hydrophobic molecules (steroid hormones, retinoids, and lipopolysaccharides) in circulation. LCN2 plays an essential role in normal physiology and disease, in several organs such as liver, renal, brain, muscle, and lung, including malignant tumors, and has been extensively studied as a biochemical marker [142].

Elevated quantities of NGAL in plasma were connected with breast cancer and associated with inflammatory reactions [143]. A systematic meta-analysis illustrated that NGAL is a potential diagnostic biomarker of breast cancer.

LCN2 has been implicated in the evolution of breast cancer, through various mechanisms, such as EMT, angiogenesis, MMP-9 upregulation, apoptosis inhibition, and iron accumulation [144,145]. LCN2 has also been correlated with poor prognostic factors such as high histological grade, ER and PR negativity, tumor relapse, as well as poor patient survival [146]. Recently it was described elevated expression of LCN2 in inflammatory breast carcinoma which advanced tumor growth, skin invasion, and metastasis [147].

In TNBC cells, autocrine excretion of LCN2, incited by loss of the tumor suppressor gene HIC1, activated the AKT pathway and caused tumor progression. The authors suggested that the HICI-LCN2 axis may be used as a prognostic biomarker for targeted TNBC therapy [89].

LCN2 has also been investigated as a therapeutic target for TNBC. One group plotted a new liposomal small interfering RNA (siRNA) delivery method to target TNBC via ICAM-1. Efficient Lcn2 knockdown by this ICAM-1-targeted, LCN2 siRNA- encapsulating liposome (ICAM-Lcn2-LP) led to a significant downregulation of VEGF in MDA-MB-231 cells, which reduced angiogenesis both in vitro and in vivo [90]. Lately, an engineered PEGylated liposomal system was developed, encapsulating lipocalin 2 small interfering RNA (LCN2 siRNA) for targeting MBC cell line MCF-7 and TNBC cell line MDA-MB-231. OCT-Lcn2-Lipo also reduced VEGF-A as well as the endothelial cell (HUVEC) migration levels, and thus exhibited in vitro anti-angiogenic effects. [91]. Others targeted LCN2 using CRISPR/Cas9 which resulted in reduced cancer cell development and raised the susceptibility of MDA-MB-231 cells to cisplatin. Moreover, LCN2 depletion adequately promoted erastin-treated ferroptosis in MDA-MB-231 cells. Suppression of LCN2 has been considered an advantageous approach for sensitizing MDA-MB-231 to ferroptotic cell death [92].

Finally, BMP2, a known protumorigenic ligand in breast cancer, impressively suppressed LCN2 mRNA and protein levels in doxorubicin-resistant 4T1 cells [148].

The results concerning Lipocalin-2 expression and TNBC are summarized in Table 1.

### 4.6. Apelin

Apelin is 9-kDa peptide, first described in 1998 as the endogenous ligand of the orphan G protein–coupled receptor APJ. Ten years later a putative role for apelin in obesity was described, mainly through the promotion οf angiogenesis in adipose tissue. Apelin is upregulated by hypoxia and insulin and reduces dramatically plasma glucose by enhancing glucose utilization in peripheral tissues. Therefore, apelin probably will be considered an important target for the treatment of obesity-related insulin resistance [149].

In MCF-7 breast cancer cells, apelin initiated cell growth and invasion along the ERK1/2 pathway [123,150], and activated tumor angiogenesis [124,151]. High apelin immunohistochemical expression has been demonstrated in breast cancer and was correlated with clinicopathological factors such as tumor size, stage, histological type, lymph node status, survival [152,153], and reduced neoadjuvant chemotherapy response [154].

Recently, it was highlighted that the application of apelin to lean mice increased TNBC growth and generated brain metastases. Τhe authors next proved that the apelinergic antagonist F13A could reduce TNBC growth and be a novel therapeutic strategy for TNBC in obese conditions [93].

Finally, apelin has been considered as an immunosuppressor hormone, expressed in TME. Thus, suppression of apelin-related protumor actions may improve the therapeutic results of cancer immunotherapy. Therapeutic combination of ML221 plus DC vaccination, reduced tumor cell proliferation (*p* < 0.0001), prevented lung metastasis (*p* < 0.0001), and increased survival (*p* < 0.01). Furthermore, succession therapy raised Th1 cells while reducing the Th2 cells in the spleen and serum levels of IL-10 [155].

The data about Lipocalin-2 expression and TNBC are shown in Table 1.

### 4.7. Chemerin

Chemerin is a new adipokine that is generated by adipose tissue and liver, advocates adipocyte differentiation, and serves as a chemoattractant for macrophages and other immune cells [156]. Chemerin and its main receptor, ChemR23, are expressed by adipocytes, and upregulated during differentiation from bone marrow mesenchymal stem cells (BMSC). Chemerin levels have been proved higher in obesity, diabetes, and NAFLD and it seems to activate insulin resistance in skeletal muscle, the primary site of peripheral insulin resistance [157].

Chemerin exhibits pro- or anti-tumor responses in many tumor categories. As far as breast cancer is concerned, elevated chemerin expression in malignant vs adjacent normal breast tissue supported the notion that chemerin is an indicator of poor prognosis in breast cancer patients [158]. Others have supported the concept that chemerin may be a promising immunotherapeutic approach as elevated chemerin expression within TME of breast cancer tissue suppressed growth by enrollment of immune cells, notably NK and T cells [159].

Recently it was described that chemerin administration substantially constrained the growth, invasion, and metastasis of breast cancer cells independently of TGF-β and IGF-I [122]. Specifically, chemerin repressed tumor development in MCF-7 breast cancer cell-injected mice and decreased the osteolytic lesions ensuing from intratibial inoculation of MDA-MB-231 cells. Generally, chemerin impedes the evolution of breast cancer cells and hampers bone destruction caused by cancer cells by inhibiting osteoclast formation and activity [122].

How chemerin improves clinical outcomes through engagement of leukocytes via the receptor CMKLR1 to the role of chemerin receptor GPR1 in tumors has not been extensively researched. Thus, the researchers elected peptide LRH7-G5, which downregulated chemerin/GPR1, as an antagonist of GPR1 [123]. Therapeutic application of this peptide actually compressed TNBC cell growth, establishing GPR1 as an essential target for TNBC progression.

The results that describe the inhibitory role of chemerin in TNBC are summarized in Table 2.

### 4.8. Oncostatin M

Oncostatin M (OSM) is a member of the IL-6 superfamily with unique functions in adipocytes and adipose tissue homeostasis. The OSM can interact with two receptor compounds. The type I comprises the LIF receptor and gp130 (gp 130/LIFRβ) and the type II the OSM receptor and gp130 (gp130/OSMRβ). Elevated expression of OSM and its receptor in adipose tissue is related to systemic metabolic dysfunction such as obesity and insulin resistance [160]. It has also been suggested that high levels of OSM derived from activated immune cells operate on neighboring adipose cells to inhibit adipocyte differentiation and to raise proinflammatory activities in adipocytes [161].

In neoplastic conditions OSM has been considered as an immunoregulator of various cancers. Whereas earlier studies determined OSM as a cancer inhibitor, plentiful new studies have argued for the significance of OSM as a dynamic activator of cancer cell de-differentiation, which particularly alters the TME and results in the acquisition of CSC properties [96]. Overexpression of OSM and its receptor has been disclosed in numerous malignant tumors including breast cancer [162]. These investigations suggested that OSM and its corresponding signaling routes should be used for future therapeutic targeting.

However, the main question, whether OSM signaling has anti-tumor or pro-tumor effects, still remains to be elucidated. JAK/STAT and MAPK pathways are mainly involved in the OSM-mediated signaling [163]. It seems that the inhibitory or reciprocal OSM/STAT3/SMAD3 cooperation might explain the discrepancies between the studies, supporting the need for further work [162].

Recently it was shown that a non-cytotoxic dose of IFN-β can repress OSM-mediated CSC properties in TNBC cells, through downregulation of SNAIL which is driven by OSM/STAT3/SMAD3/TGF-β axis [163]. SNAIL is a major contributor in mesenchymal/CSC de-differentiation program. Collectively, the results indicate the conflicting roles of the TME cytokines IFN-β and OSM in regulating CSC plasticity in TNBC [96].

Lately it was demonstrated that a group of genes were incited by miR551b-3p, named as “Oncostatin signaling module” [95]. Mature miR551b-3p moves from the cytoplasm to nucleus assisted by IPO8 and activates STAT3. As a result, STAT3 coordinates the expression of OSM receptor and IL31 receptor-α (IL31RA) as well as their ligands OSM and IL31. Anti-miR551b-3p molecule reduced tumor growth as well as migration and invasion of breast cancer cells.

Analysis of the TCGA data set using the UALCAN portal demonstrated that OSM is highly deliberated in the TNBC subtype of breast cancer in comparison to the other molecular subtypes [94]. Additionally, using the KM plotter survival analysis portal it was demonstrated that higher expression of OSM in ER-negative patients was associated with poor outcomes [95].

The above data confirm that breast cancer is a highly heterogenous and complex disease and many matrix-associated molecules simultaneously moderate different oncogenic networks contributing to the complicated architecture of cancer-related pathways.

The studies that describe the promoting role of OSM in TNBC are presented in Table 1.

### 4.9. Osteopontin

Osteopontin (OPN) is an abundant non-collagenous phosphoglycoprotein of the bone ECM, composed of three splicing variants (-a, -b, and -c). OPN is secreted predominantly by osteoblasts and osteocytes and coordinates matrix mineralization and bone cell (osteocyte and osteoclast) junction. OPN is also expressed by divergent cell types (epithelial cells, T-cells and macrophages) [164] and interacts with several cell surface receptors (integrins, CD44, etc.) [165] contributing in a broad range of normal and clinical conditions [166,167]. In recent years OPN was connected with adipose tissue inflammatory situations, obesity, and insulin resistance through monocyte chemotaxis and macrophage proliferation and differentiation [168]. Regarding neoplastic conditions, OPN is overexpressed in many cancers and was considered as a robust therapeutic target [169]. OPN was established in breast cancer as associated with metastatic potential [170]. High OPN expression in serum or tissue of breast cancer patients has been considered as negative prognostic biomarker in breast cancer and has been associated with advanced stages of the disease and poor patient outcome [170].

It has been reported that OPN overexpression is predominantly associated with triple-negative phenotype [171]. Results from investigation of large series of breast cancer patients display that OPN-t is mainly overexpressed in aggressive phenotypes (HER2+ and TN/basal-like tumors) and in particular OPN-c mRNA in TN/basal-like ones [97]. Recently, the concept that OPN exerts some of its malignancy-advancing events through EGFR activation has been supported. Studies explored the assumption that OPN-expressing TNBC cells have improved response to EGFR inhibitors such as erlotinib and that OPN could be used as a predictive biomarker for treatment response [172].

### 4.10. Other Adipokines

Cytokines, including IL-6 and tumor necrosis factor (TNF)-α released by adipocytes, increase aromatase expression and estrogen synthesis in peripheral organs and tissues, including breast [173]. TNF-α also generates IL-6 in MDA-MB-231 cells through ERK1 stimulation [98]. IL-6 and CCL5 advocate crosstalk between TNBC and lymphatic vessels and enhance TNBC tumor growth and metastasis [99]. IL-6 secreted by adipocytes triggers IL-6/STAT3 axis and drives the acquisition of EMT properties in breast cancer cells [174]. Adipocytes enhanced MDA-MB-231 cancer cell invasiveness, through CCL5 signaling, which negatively correlated with OS [60]. IGF-1, produced by adipocytes, binds to IGFI on cancer cells and endorses cancer cell proliferation through PI3K/AKT and MAPK systems [18], whereas inhibition of IGF-1R abolishes the tumor promoting effect of adipocytes on breast cancer cells [60]. Recently it has been shown that in TNBC cells, the IGF1/IGF1-R signaling promotes the activation of the FAK-YAP cascade that results in the proliferation of TNBC cells [100].

Adipose stem cells (ASCs) are a key participant of breast cancer microenvironment which harbor breast cancer cells from the host immune responses. A recent study reported that the VEGF, IL-8, HGF, and IGF-I expression in ASCs resided in breast cancer tissues, and was twofold higher in patients than controls. Data concluded that the existence of native ASCs within breast tissue may sustain breast cancer growth [175]. In accordance with that, another study proposed that a molecular crosstalk between ASCs and breast cancer cells play a crucial role in radioresistance. It was shown that the IGF-IR expression was substantially elevated after breast cancer cells radiation and suggested that breast cancer cells possibly recruit ASCs, which produce IGF-I to enhance radioresistance [176].

HGF is a mitogenic factor released by adipose cells. Its receptor, c-MET, is expressed at high levels in breast cancer cells situated at the interface with adipocytes, highlighting the importance of stromal–tumor cell interactions in mammary tumor growth [101]. In TNBCs, elevated levels of the c-MET receptor predict poor clinical outcome [102]. Recent studies have concluded that ASCs assist breast cancer recurrence and progression via the HGF/c-MET pathway [177].

The last section of Table 1 shows the results that describe the promoting role of other adipose tissue associated cytokines in TNBC.

## 5. Conclusions

**Figure 1:**
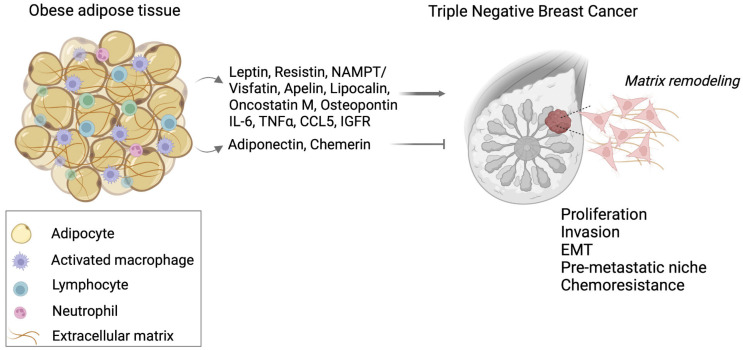
Schematic representation of correlation among obesity and TNBC. Adipocyte accumulation results in the secretion of inflammatory factors (i.e., ILs, TNFα), adipokines (i.e., leptin, resistin, apelin), and matrix mediators (i.e., IGFR), which establish a pro-tumorigenic microenvironment that boosts TNBC cells’ aggressive properties, including proliferation, invasion, and EMT. These contribute to metabolic modifications of the tumor microenvironment, initiating a pre-metastatic niche that contributes to breast cancer progression and acquired drug resistance. Created with Biorender.com. Abbreviations: EMT, epithelial-to-mesenchymal transition; IGFR, insulin-like growth factor receptor; IL, interleukin; TNF, transforming growth factor.

The present review highlights the association between adipokine expression and TNBC. Although data suggest that increased BMI may be a significant factor for TNBC development, the pathogenetic procedures through which adipose tissue-associated factors mediate TNBC development and progression does not appear to be clearly defined. The crosstalk between adipose tissue and TNBC cells has been explored in several in vitro studies in which it was demonstrated that adipose tissue associated mesenchymal and stem cells promote tumor growth and invasion of breast cancer cells. During the development of obesity, the hypoxic TME in breast promoted the expression of an altered profile of inflammatory cytokines and hormones called adipokines which establish a chronic low-grade state of inflammation and predispose to the development of metabolic morbidities as well as cancer. Probable mechanisms through which adipokines induce carcinogenesis, include initiation of EMT, matrix degradation through elevated expression of MMPs, induction of angiogenesis, immunosuppression, chemoresistance, as well as secretion of paracrine factors favoring metastatic spread (Figure 1).

Adipokines overexpressed in TNBC include leptin, resistin, NSMPT, LCN2, and apelin, whereas adiponectin and chemerin are significantly downregulated and their protective role against breast cancer has been suggested. Of particular interest, converging data suggest that an increased leptin:adiponectin ratio is strongly associated with with TNBC rather than ER positive breast cancer.

Due to the lack of effective therapies and the worse prognosis of TNBC, the consideration of molecular mechanisms of adipokine expression and their interplay offer novel treatment options with new pharmacological therapies that target critical, adipose associated molecular pathways. Many advances have been made on this field and new therapeutic approaches with adipokine inhibitors have been proved effective in preclinical studies. However, the diverse actions of known and newly identified adipokines in the obese tumor microenvironment must be examined carefully, and the beneficial and toxic effects of adipokine targeting drugs, alone or in combination with conventional chemotherapy, need to be investigated in-depth in future studies.

## Figures and Tables

**Table 1:** Expression of adipokines with pro-tumor activity in TNBC.

Adipokines/Receptors	Expression/Actions in TNBC	References
Leptin/LEPR	Leptin and LEPR are essentially overexpressed in TNBC	[72]
	A significant crosstalk between leptin and IGF-I increase the activation of EGFR and LEPR and drive TNBC progression	[73]
	Leptin induces BC stemness and resistance to chemotherapy	[75]
	Elevated leptin levels develop EMT	[77]
	Leptin receptor antagonist peptide Allo-aca inhibited leptin-induced proliferation of MDA-MB-231 cells	[78]
	Leptin secreted by obesity altered adipose stem cells induced the EMT and CSC reprogramming	[79]
	Reduced IHC expression of LEPR was correlated with ER-status and TN subtype	[80]
	Leptin induces apoptosis in TNBC cells when used in combination with cAMP elevating agents	[81]
Resistin	Resistin increases the malignant potential of MCF-7 and MB-231 cells through EMT initiation and stemness	[82,83]
	Higher expression of both TAZ and resistin in adipose tissue of TNBC tumors correlated with higher stage and poor prognosis, giving the idea of targeted therapy	[84]
NAMPT/visfatin	NAMPT inhibits autophagy and apoptosis and induces cell survival and invasiveness through mTOR activation in TNBC cells	[85]
	The combined use of NAMPT inhibitor FK866 with olaparib inhibited TN breast tumor growth in vivo	[86]
	Treatment with the small molecule KPT-9274, inhibitor of PAK4 and NAMPT, abrogates TNBC cell proliferation and eventually leads to cell death	[87]
	NAMPT inhibitor FK866 combined with FX11 (lactate dehydrogenase A inhibitor) and paclitaxel caused significant growth restriction of MDA-MB-231 cells	[88]
Lipocalin2 (NGAL)	In TNBC cells, secretion of LCN2, induced by HIC1 loss, activated the AKT pathway and caused tumor progression	[89]
	Lcn2 knockdown by ICAM-LCN2-LP led to a significant reduction of VEGF in MDA-MB-231 cells, which led to reduced angiogenesis both in vitro and in vivo	[90]
	Silencing of Lcn2 mRNA by OCT-Lcn2-Lipo display anti-angiogenic results in MCF-7 and MDA-MB-231 cells by diminishing VEGF-A and endothelial migration	[91]
	Targeting Lcn2 by CRISPR/Cas9 reduced cancer cell malignant potential and increased cell susceptibility of MDA-MB-231 cells to cisplatin	[92]
Apelin	The administration of apelin to lean mice elevated TNBC growth and brain metastases. The apelinergic antagonist F13A could reduce TNBC growth and be a novel therapeutic strategy for TNBC in obese conditions	[93]
Oncostatin M	OSM is thoroughly expressed in the TNBC subtype in comparison with other molecular breast cancer subtypes	[94]
	The KM plotter survival analysis portal demonstrated that higher expression of OSM ER-negative patients was associated with poor outcomes	[95]
	TME cytokines OSM and IFN-b express antagonistic roles in CSC plasticity coordination in TNBC	[96]
	miR551b-3p named as “Oncostatin signaling module” translocates from the cytoplasm to nucleus and upregulates the expression of OSM receptor IL31 receptor as well as their ligands OSM and IL31	[95]
Osteopontin	OPN mRNA is upregulated in triple negative/basal like tumors	[97]
Other	TNF-a induces IL-6 in MDA-MB-231 cells via ERK1 activation	[98]
	IL-6 and CCL5 promote TNBC tumor growth via cancer cell-lymphatic vessels cross talk	[99]
	Adipocytes enhanced MDA-MB-231 cancer cell invasiveness, through CCL5 signaling, which negatively correlated with OS	[60]
	The IGF-I/IGF-IR signaling promotes the FAK-YAP cascade activation and triggers TNBC growth	[100]
	Hepatocyte growth factor (HGF) is a mitogenic factor released by adipocytes and its receptor c-met is expressed at high levels on breast cancer cells, at the adipose-cancer interface, highlighting the importance of stromal–tumor cell interactions in breast cancer growth. In TNBCs, elevated levels of the MET receptor predict poor clinical outcome	[101,102]

**Table 2:** Expression of adipokines with anti-tumor activity in TNBC.

Adipokines/Receptors	Expression/Actions in TNBC	References
Adiponectin/ AdipoR1/AdipoR2	Reduced adiponectin: leptin is correlated with the diagnosis of TNBC	[116]
	In ER/PR-negative BC cells, it inhibits cell growth, invasion, migration, and vascular proliferation and induces apoptosis and autophagic cell death	[117,118]
	Normal adiponectin amounts significantly suppress the proliferation of MDA-MB-231 cancer cells	[119]
	Diminished adiponectin is thoroughly correlated with TNBC development and progression, regardless of obesity and insulin resistance	[120]
Chemerin	Chemerin restricts the growth and invasion of breast cancer cells and prevents bone loss resulting from MDA-MB-231 cell growth	[122]
	Treatment with peptide LRH7-G5 significantly decreased TNBC cell growth demonstrating chemerin/GPR1 as a novel therapeutic target for TNBC	[123]
